# Metadherin Contributes to the Pathogenesis of Diffuse Large B-cell Lymphoma

**DOI:** 10.1371/journal.pone.0039449

**Published:** 2012-06-29

**Authors:** Xueling Ge, Xiao Lv, Lili Feng, Xiaoqian Liu, Junming Gao, Na Chen, Xin Wang

**Affiliations:** 1 Department of Hematology, Provincial Hospital Affiliated to Shandong University, Jinan, People’s Republic of China; 2 Department of Hematology, Yantai Yuhuangding Hospital, Affiliated Hospital of Medical College, Qingdao University, Yantai, People’s Republic of China; 3 Department of Internal Medicine, Shandong Police Officer General Hospital, Jinan, People’s Republic of China; 4 Institute of Diagnostics, Shandong University, Jinan, People’s Republic of China; Sun Yat-sen University Medical School, China

## Abstract

**Background:**

Metadherin (MTDH) has been demonstrated as a potentially crucial mediator of various types of human malignancies. However, the expression and role of MTDH in diffuse large-B-cell lymphoma (DLBCL) have not been reported yet. This study aimed to illuminate the role of MTDH in the pathogenesis of DLBCL.

**Methodology/Principal Findings:**

A remarkable elevation of MTDH on mRNA level was detected in DLBCL tissues by quantitative polymerase chain reaction (PCR). Using Western-blot analysis we found that the expression of MTDH protein was significantly upregulated in DLBCL cell lines and DLBCL tissues compared with peripheral blood mononuclear cells (PBMCs) from healthy samples and tissues from patients of reactive hyperplasia of lymph node. The results showed high expression of MTDH in 23 of 30 (76.67%) DLBCL tissues by using immunohistochemical analysis and the over expression of MTDH was strongly correlated to the clinical staging of patients with DLBCL (P<0.05). Furthermore, the finding suggested that the increase of MTDH in DLBCL cells could distinctly enhance cell proliferation and inhibit cell apoptosis; meanwhile, inhibition of MTDH expression by specific siRNA clearly enhanced LY8 cell apoptosis. Upregulation of MTDH elevated the protein level of total β-catenin and translocation of β-catenin to the nucleus directly or indirectly. Knockdown of MTDH decreased the level of total, cytoplasmic β-catenin and reduced nuclear accumulation of β-catenin protein. This indicated that the function of MTDH on the development of DLBCL was mediated through regulation of Wnt/β-catenin signaling pathway.

**Conclusions/Significance:**

Our results suggest that MTDH contributes to the pathogenesis of DLBCL mediated by activation of Wnt/β-catenin pathway. This novel study may contribute to further investigation on the useful biomarkers and potential therapeutic target in the DLBCL patients.

## Introduction

Diffuse large-B-cell lymphoma (DLBCL) is an aggressive malignancy of mature B lymphocytes and is the most common type of lymphoma in adults [1], [2], accounting for 25% to 30% of adult non-Hodgkin lymphoma in the West. It is even more prevalent in developing countries [3], [4]. DLBCL is an aggressive disease with variable biological and cytogenetic features, as well as clinical outcomes. About 40% of patients with DLBCL achieve long-term survival after anthracycline-based chemotherapy [5]. However more intensive chemotherapy were failed to demonstrate additional benefits because of more toxicity [6]. In the past few years remarkable progress has been made in understanding the biological heterogeneity of DLBCL and in improving survival for DLBCL patients with combinations of chemotherapy and immunotherapy. The introduction of antilymphoma monoclonal antibodies, notably rituximab, in combination with chemotherapy has significantly improved survival outcomes of patients with DLBCL [7], [8]. Nevertheless it is still fatal in the half of patients [9]. Most lymphoma probably activates more than one survival signaling pathway; therefore, clinical studies should be promoted with combination of multiple molecular targeted agents. The use of single agent will result in a tolerant process where the tumor cells will develop the signaling pathways not currently inhibited. The development of biomarkers, which are able to select patients who will respond to one or several of these targeted therapies, is important too [10]. In view of this miserable illustration, further investigating specific biomarkers and cellular signaling pathways, understanding molecular pathogenesis of DLBCL and developing more targeted and effective treatments are indispensable for significantly increasing the survival and alleviating the suffering of patients.

Metadherin (MTDH, also known as astrocyte elevated gene-1/AEG-1 and Lyric) was first cloned as an HIV- and TNF-α–inducible gene in primary human fetal astrocytes (PHFAs) [11], [12]. Human MTDH encodes a 582-amino acid protein with a predicted molecular mass of 64 kDa. Antibodies against MTDH frequently detect multiple proteins with molecular weights ranging from 75–80 kDa to 20 kDa, possibly due to alternative splicing and/or posttranslational modification [12]–[14]. In recent years, MTDH, involved in aberrant proliferation, survival, and increased migration, invasiveness, and metastasis of tumor cells, has been demonstrated as a potentially crucial mediator of various types of human malignancies. Expression analysis revealed that MTDH expression is significantly higher in melanoma, breast, esophageal, gastric, hepatocellular, endometrial and prostate cancers, renal cell carcinoma, neuroblasoma and malignant glioma cell lines compared with their normal counterparts [12], [15]–[23]. These observations in cell lines have been confirmed in tumor samples of patients mainly by immunohistochemistry or gene expression profiling [15], [16], [18]–[27]. MTDH promotes tumor progression by modulating multiple oncogenic signaling pathways, such as NF-κB, PI3K/Akt and Wnt/β-catenin pathways [28], [29]. In HeLa cells and human malignant glioma cells treated with tumor necrosis factor-α (TNF-α, which induces MTDH overexpression), MTDH translocates into the nucleus where it interacts with the p65 subunit of NF-κB and upregulates NF-κB-induced gene expression [29], [30]. IL-8, one of NF-κB downstream genes, positively regulated angiogenesis and metastasis and inhibition of NF-κB blocked MTDH-mediated anchorage-independent growth and invasion by HeLa cells [29]. A second signaling pathway regulated by MTDH is PI3K/Akt pathway. Interestingly, this pathway is not only activated by MTDH but also plays an important role in regulating MTDH expression [31]. MTDH overexpression promotes phosphorylation of Akt and GSK3β [32], and affects many additional Akt downstream genes that are crucial for cellular proliferation, survival and apoptosis. MTDH knockdown induces apoptosis of prostate cancer cells through the attenuation of Akt activity and upregulation of FOXO3a activity [18]. More recently, MTDH has been found to connect with Wnt/β-catenin pathway in hepatocellular carcinoma cells through the activation of the Raf/MEK/MAPK branch of Ras signaling pathway, leading to β-catenin nuclear translocation and upregulation of different target gene expressions [16]. Inhibition of MTDH expression by specific siRNA clearly decreased the level of β-catenin and it may play a role in Wnt/β-catenin-mediated gastric cancer progression [15]. These findings demonstrate that MTDH is both a potentially biomarker of tumor malignancy and a crucial integration factor of multiple oncogenic signaling pathways. Wnt/β-catenin signaling pathway not only acts a significant part in embryonic development and in maintenance of organs and tissues in adults [33], [34] but also involves in the pathogenesis of a range of disease including many kinds of carcinomas [35]. Wnt/β-catenin pathway plays an important role in progression of several subtypes of lymphoma such as Epstein-Barr Virus (EBV) -positive Burkitt’s lymphoma (BL) [36], mantle cell lymphoma (MCL) [37], [38], cutaneous lymphoma [39], extranodal marginal zone lymphoma [40], DLBCL [41], [42], [43], among others [44], [45]. Since aberrant activation of Wnt/β-catenin pathway is probably important for lymphoma development and progression including DLBCL, we hypothesize that MTDH is in close contact with Wnt/β-catenin pathway in DLBCL and contributes to the pathogenesis of DLBCL.

For the first time, our present manuscript focuses on illuminating the role of MTDH and the relationship between MTDH and Wnt/β-catenin pathway in the pathogenesis of DLBCL. We demonstrate the overexpression of MTDH and β-catenin in DLBCL and the effect of MTDH expression on biological behavior of DLBCL cell lines. We also provide evidences for the link between MTDH and Wnt/β-catenin pathway in DLBCL.

## Results

### MTDH is Overexpressed in DLBCL

**Figure 1:**
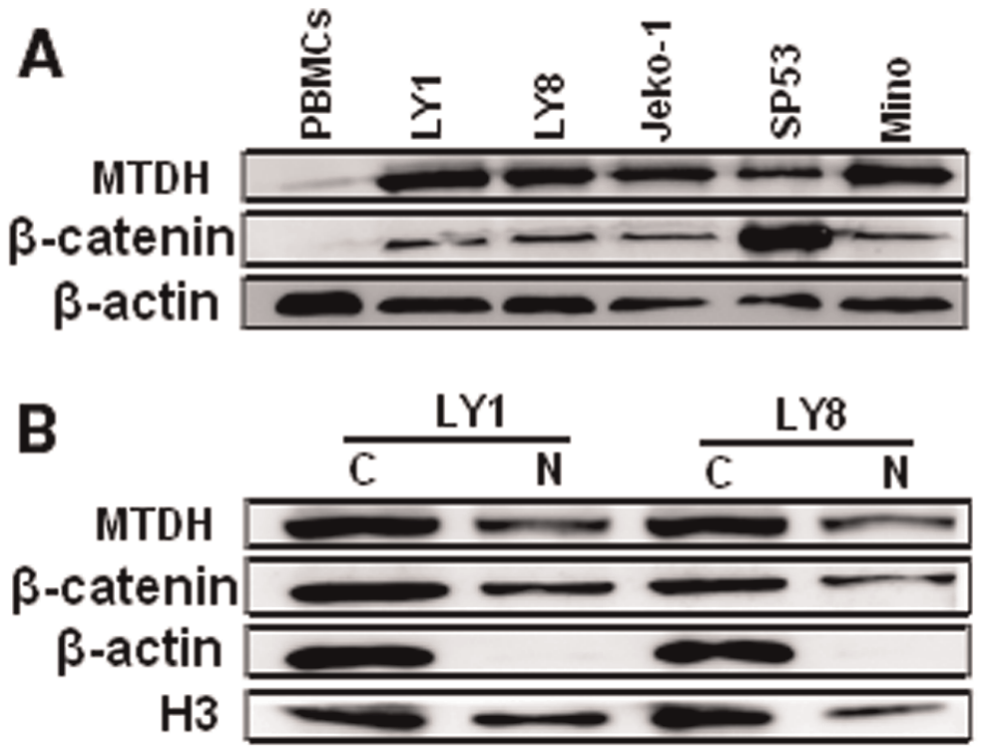
MTDH and β-catenin are overexpressed and nucleic localization in DLBCL cell lines. (A) Expression of MTDH (75 kDa) and β-catenin (94 kDa) were detected in the indicated cell lines. PBMCs represent peripheral blood mononuclear cells from healthy samples. Expression of β-actin was used as loading control. (B) Subcellular protein fractionation using the cell lysates of 2 DLBCL cell lines revealed that MTDH and β-catenin were localized in the nucleus (N) and possibly also in the cytoplasm (C) and. The expression of β-actin in the cytoplasm and H3 in the nucleus served as controls for the efficiency of subcellular fractionation.

**Figure 2:**
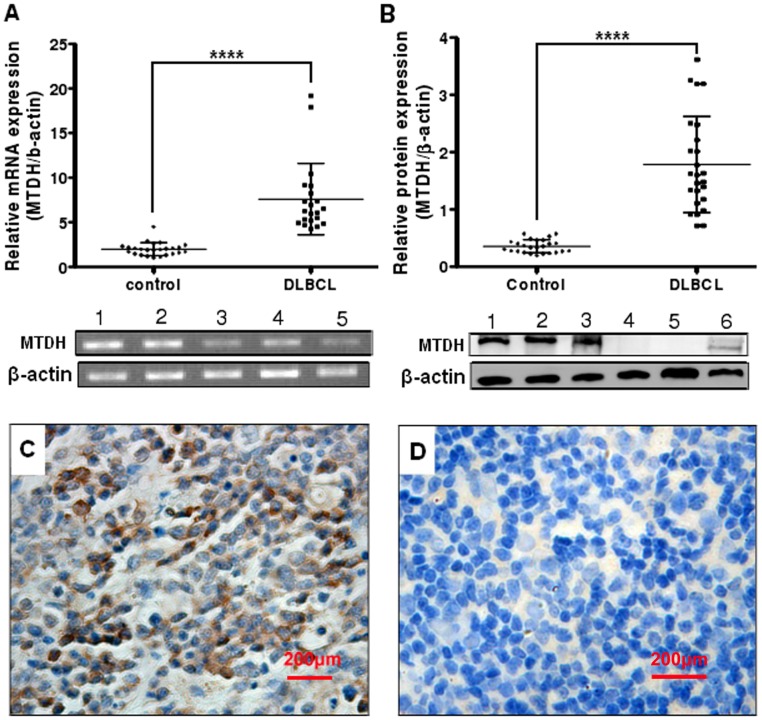
MTDH is overexpressed in DLBCL tumor samples. (A) Real-time quantitative PCR analysis of MTDH expression in DLBCL (n = 21) and control (n = 25). PCR products were confirmed as a single product at the desired size on agarose gels (1–3: DLBCL; 4–5: Control). Control, reactive hyperplasia of lymph node. β-actin was used as a loading control. (B) Expression of MTDH protein (75 kDa) in DLBCL (1–3) and reactive hyperplasia of lymph node tissues (4–6). Expression levels were normalized with β-actin. (C) Detection of MTDH expression in DLBCL by IHC. (D) Detection of MTDH expression in reactive hyperplasia of lymph node by IHC. Original magnification, ×400. Points indicate mean of triplicate determinations, bars, SD. ****p<0.0001 versus control.

Expression of MTDH was detected by Western blot, with the single band size of 75 kDa, in peripheral blood mononuclear cells (PBMCs) from healthy samples, human DLBCL cell lines LY1 and LY8, and MCL cell lines Jeko-1, Mino, and SP53. The protein level of MTDH was much lower in PBMCs from healthy samples compared with all the human DLBCL and MCL cell lines (Figure 1A). Moreover MTDH was found in the nuclear fractions, and possibly also in the cytoplasmic fractions, in both 2 DLBCL cell lines (Figure 1B). To examine whether the expression of MTDH in DLBCL tissues is clinically correlated with DLBCL pathogenesis, comparative analysis of MTDH expression was conducted on patient samples and inflammatory lymph node tissues through real-time quantitative PCR, immunohistochemistry and Western blot. MTDH mRNA was found to be differentially overexpressed in DLBCL samples, whereas it was weakly expressed in reactive hyperplasia of lymph node tissues (P<0.0001, Figure 2A). Western blot analysis revealed that DLBCL exhibited significantly higher levels of MTDH protein expression compared to levels in their counterparts (P<0.0001, Figure 2B).

To further determine whether MTDH protein overexpression is associated with clinicopathological characteristics of DLBCL, a cohort of paraffin-embedded, archived DLBCL tissues (n = 30) were examined by immunohistochemical staining with an antibody against human MTDH. As shown in Figure 2C, significant MTDH staining was observed in DLBCL samples, while only little or rather non-expression of MTDH was detected in the 15 reactive hyperplasia of lymph node tissues (Figure 2D). Among the 30 DLBCL samples, only 7 negative for MTDH and the remaining 23 (76.67%) showed variable levels of MTDH. MTDH expression was detected predominantly in the cytoplasm and occasionally in the nucleus. Taken together, these observations suggested that MTDH upregulation was associated with the pathogenesis of DLBCL.

### Relationship between MTDH Upregulation and the Clinicopathological Characteristics of DLBCL

Statistical analyses were done to examine the correlation between the expression of MTDH protein by immunohistochemistry analysis and the clinical features of DLBCL. As shown in Table 1, there was no correlation between MTDH expression and patient age (P = 0.204), gender (P = 1.000) or B symptoms (P = 1.000). Nevertheless, the expression of MTDH protein analyzed by immunohistochemical staining was strongly correlated to the clinical staging of patients with DLBCL (P<0.05), which was further confirmed by the Spearman rank correlation analysis. Spearman correlation of MTDH expression to clinical staging was 0.507 (P = 0.004). Together, these results indicate that the overexpression of MTDH is associated with DLBCL clinical progression.

**Table 1:** Correlation between MTDH expression by immunochemistry analysis and the clinicopathological characteristics of DLBCL patients.

Characteristics	MTDH expression		Fisher’s Exact Test P value
	Negative no.(%)	Positive no.(%)	
Age(years)			
<60	2 (12.5)	14 (87.5)	0.204
>60	5 (35.7)	9 (64.3)	
Gender			
Male	5 (22.7)	17 (77.3)	1.000
Female	2 (25.0)	6 (75.0)	
Clinical stage			
I	4 (57.1)	3 (42.9)	0.047
II	2 (28.6)	5 (71.4)	
III	1 (20.0)	4 (80.0)	
IV	0 (0.0)	11(100.0)	
B symptoms			
Yes	3 (23.1)	10 (76.9)	1.000
No	4 (23.5)	13 (76.5)	

### Induction of MTDH Enhances Proliferation and Inhibits Apoptosis of DLBCL Cells and Knockdown of MTDH Reinforces Apoptosis of DLBCL Cells

**Figure 3:**
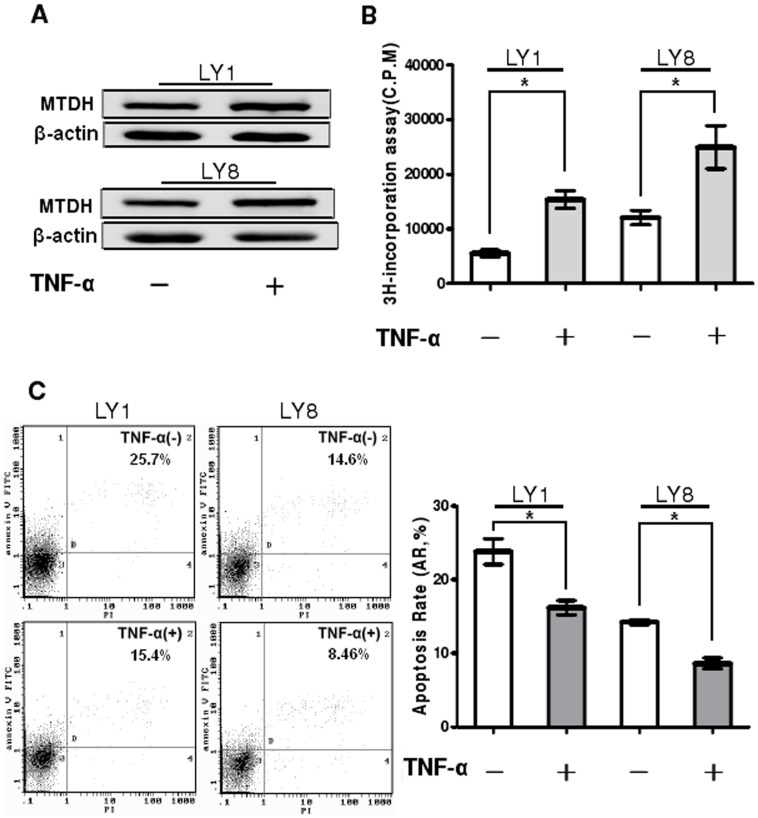
Upregulation of MTDH enhances proliferation and inhibits apoptosis of DLBCL cells. (A) LY1 and LY8 cells were either untreated or treated with 250 pg/mL of TNF-α for 48 hours. The expression of MTDH protein was analyzed by Western blot. (B) DLBCL cells were treated with TNF-α at the indicated concentration for 48 hours, and cell proliferation was determined by 3H-TdR incorporation assay. Columns indicate mean of triplicate determinations; bars, SD. (C) LY1(left panel) and LY8(right panel) cells were treated with TNF-α (250 pg/mL for 48 hours) and cell apoptosis was detected by flow cytometer. Early apoptotic cells were defined as Annexin-V-FITC-positive, PI-negative cells. Columns indicate mean of triplicate determinations; bars, SD. *p<0.05 versus control.

It is previously reported that TNF-α up-regulates MTDH expression at both the mRNA and protein level in the human cervical carcinoma cell line HeLa [29]. To verify the effect of TNF-α on MTDH expression in DLBCL cells, the protein level of MTDH in LY1 and LY8 cell lines was determined by using Western blot analysis after treatment with TNF-α for 48 hours (final concentration 250 pg/ml). As shown in Figure 3A, the expression of MTDH was up-regulated about 20% at the protein level after TNF-α treatment (P<0.05). Based on the results above, DLBCL cells were exposed to TNF-α for further studies investigating the potential functions of MTDH in these cells. The effect of MTDH on the proliferation of LY1and LY8 cell lines was accessed with ^3^H-TdR incorporation assay. The increase of MTDH could promote the proliferation of 2 DLBCL cell lines (P<0.05, Figure 3B). To determine whether the enhancing proliferation of LY1 and LY8 cells due to upregulation of MTDH was associated with the apoptosis of these cells, the effect of MTDH on the apoptosis of LY1 and LY8 was analyzed using Annexin V-FITC/PI staining followed by flow cytometry (Figure 3C). Cell apoptosis was inhibited after MTDH overexpression by exposure (48 hours) to TNF-α at the indicated concentration in comparison with untreated cells (P<0.05).

**Figure 4:**
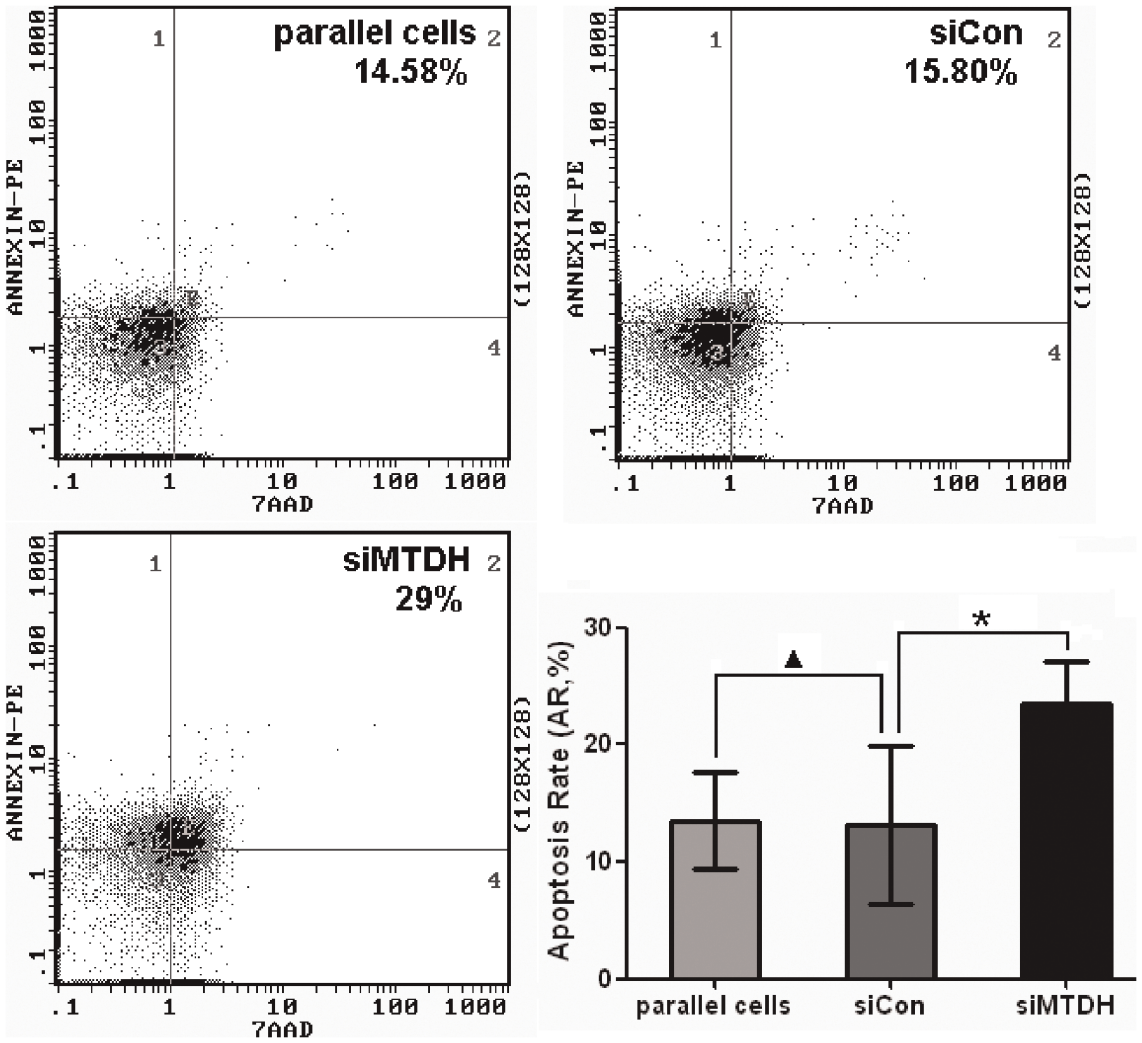
Knockdown of MTDH promotes apoptosis of DLBCL cells. LY8 cells were transfected with MTDH siRNA or negative control siRNA and cell apoptosis was detected by flow cytometer. Early apoptotic cells were defined as Annexin-V-PE-positive, 7-AAD-negative cells. Columns indicate mean of triplicate determinations; bars, SD. *p<0.05 versus control; ▴p>0.05 versus control.

To further investigate the potential function of MTDH gene in DLBCL pathogenesis, cell apoptosis was evaluated in MTDH knockdown cells (Figure S1). As shown in Figure 4, MTDH knockdown cells exhibited significantly higher apoptotic rate in comparison to the cells transfected with negative control siRNA (P<0.05).

**Figure 5:**
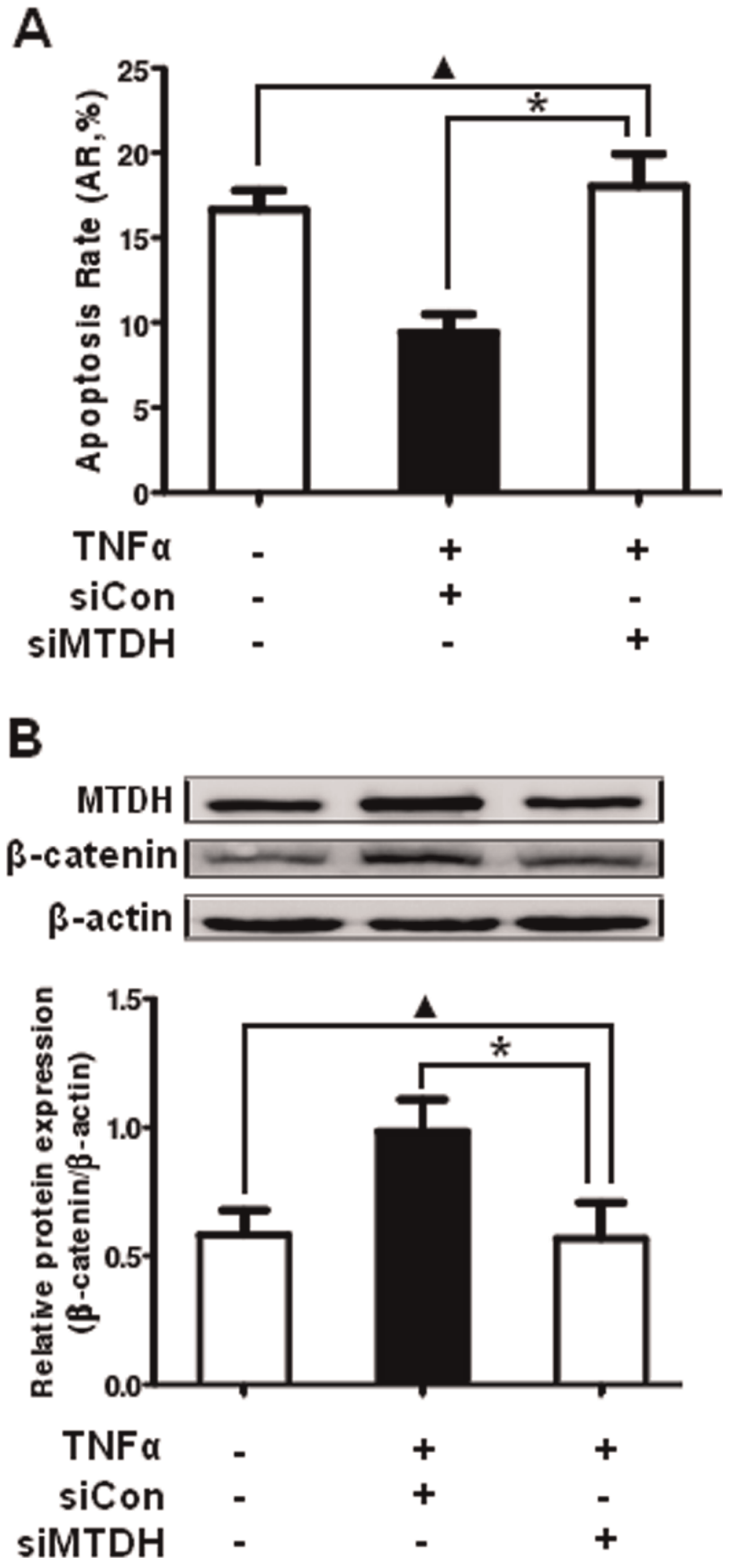
Combined action of TNF-α treatment and siRNA towards MTDH. (A) LY8 cells transfected with control siRNA and MTDH siRNA were either untreated or treated with 250 pg/mL of TNF-α for 48 hours. Then cell apoptosis was detected by flow cytometer. (B) Detection of total β-catenin proteins by Western blot analysis in transfected LY8 cell line untreated and treated with TNF-α at the indicated concentration and exposure time. Data expressed as mean±SD. *p<0.05 versus control; ▴p>0.05 versus control.

Between the LY8 cells untreated and treated with TNF-α and siRNA towards MTDH, there was little change in their apoptosis rate when the expression of MTDH was detected to be unchanged (Figure 5A, P>0.05). Besides, the cells treated with TNF-α and control siRNA showed lower apoptosis rate than the cells treated with TNF-α and MTDH siRNA (Figure 5A, P<0.05). This probably showed that the effect on the proliferation and apoptosis occurred after the expression change of MTDH rather than directly after TNF-α treatment. Taken together, these results suggested that MTDH could promote cell apoptosis and was essential for the proliferation in DLBCL cells, so that it probably plays a key role in the pathogenesis of DLBCL.

### Nuclear Localization of β-catenin in DLBCL Cells

To determine the activity of Wnt/β-catenin pathway in DLBCL, we presently assessed the expression of β-catenin, the chief downstream effector of Wnt/β-catenin pathway, in 2 DLBCL, 3 MCL cell lines. As illustrated in Figure 1A, the protein level of β-catenin was distinctly higher in the human DLBCL and MCL cell lines compared with PBMCs from healthy samples. Furthermore, since β-catenin is a transcription factor that migrates to the nucleus and mediates the activation of Wnt/β-catenin pathway [37], we assessed its nuclear localization. Making use of subcellular protein fractionation and Western blot, β-catenin was detected in the nuclear lysates, and possibly also in the cytoplasmic lysates, in 2 DLBCL cell lines (Figure 1B). The expression of β-actin in the cytoplasm and H3 in the nucleus served as controls for the efficiency of subcellular protein fractionation.

### Wnt/β-catenin Pathway is One of the Downstream Signalings Activated by MTDH in DLBCL

**Figure 6:**
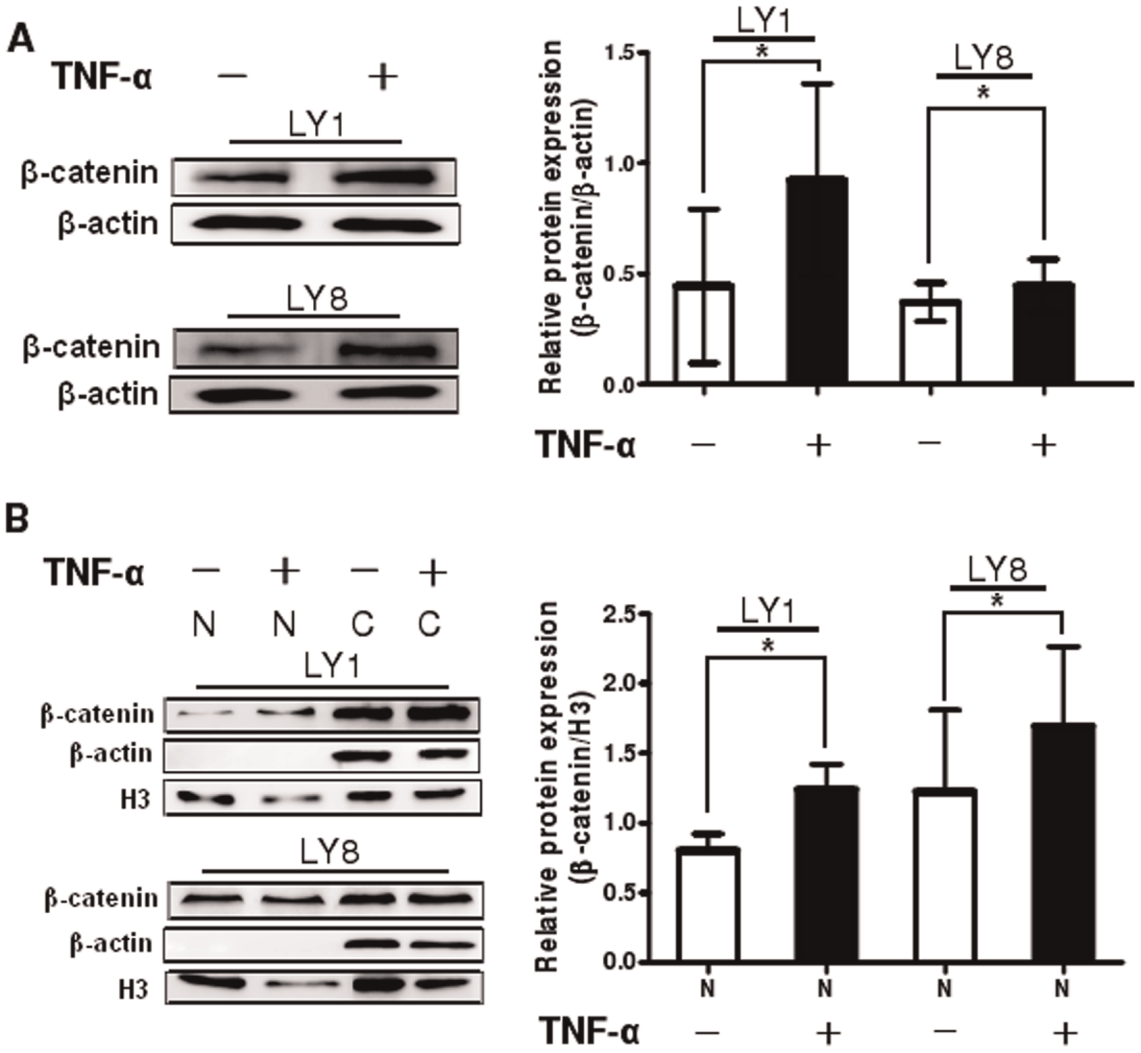
MTDH mediated aberrant activation of Wnt/β-catenin pathway in DLBCL cells. (A) Detection of total β-catenin proteins by Western blot analysis in LY1 and LY8 cell lines untreated and treated with TNF-α at the indicated concentration and exposure time. (B) Analysis of nuclear β-catenin protein expression using subcellular fractionation and Western blot. The expression o f β-actin in the cytoplasm(C) and H3 in the nucleus (N) served as controls for the efficiency of subcellular fractionation. Data expressed as mean±SD. *p<0.05 versus control.

Since Wnt/β-catenin pathway was found to be aberrant in DLBCL cells and is probably involved in the pathogenesis of DLBCL, we focused on discussing whether Wnt/β-catenin pathway is overactivated by MTDH using western blot analysis. Total β-catenin protein level was increased in TNF-α-treated cells (P<0.05, Figure 6A). The transcriptional activity of β-catenin requires its nuclear migration of β-catenin [37], therefore we checked the localization of β-catenin with subcellular protein fractionation and Western blot analysis. In the TNF-α untreated DLBCL cells β-catenin was localized both in the cytoplasm and the nucleus as described previously. After treatment with TNF-α, the protein level of nuclear β-catenin was elevated in the meantime (P<0.05, Figure 6B) following MTDH upregulation.

**Figure 7:**
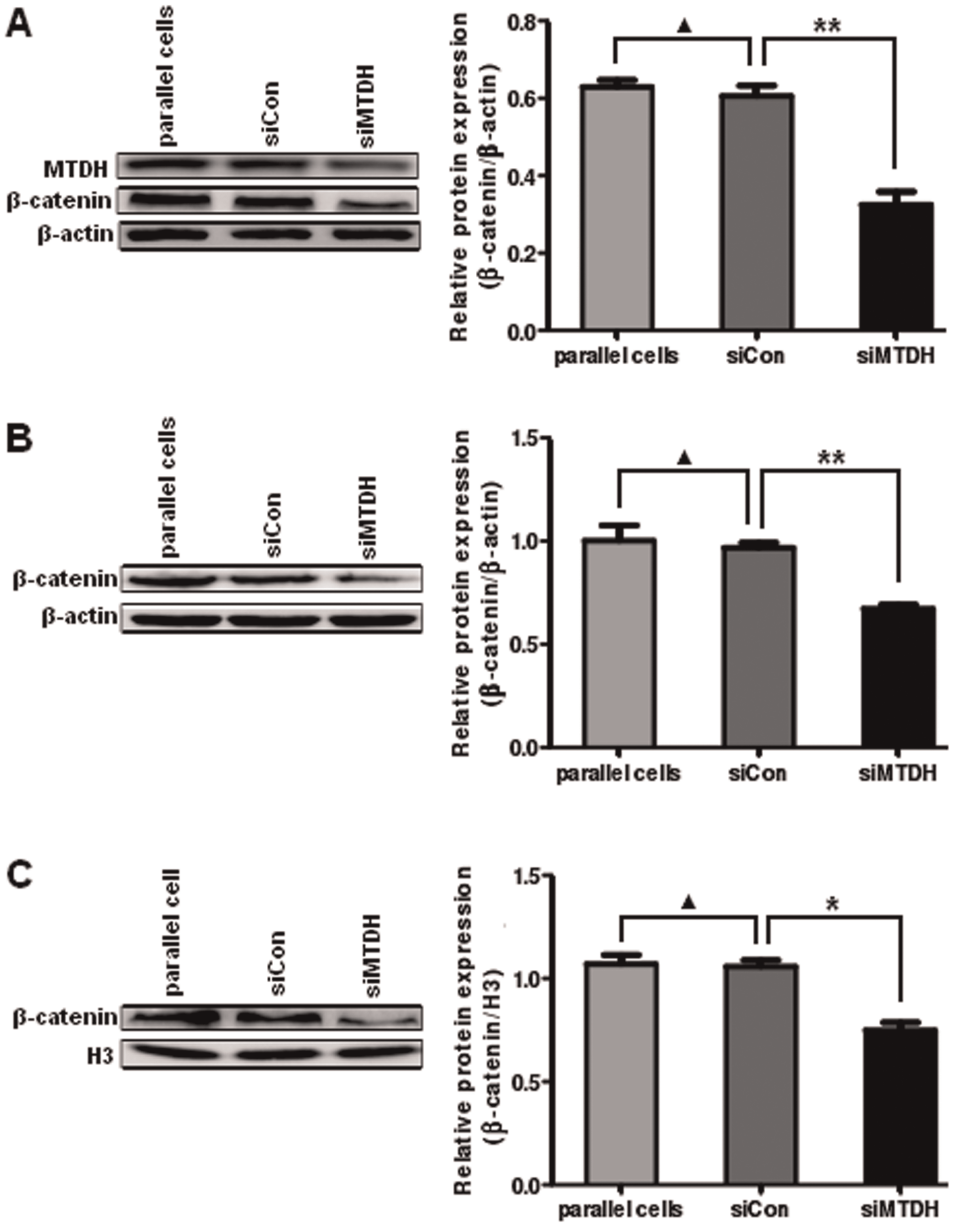
MTDH downregulation inhibits the activity of Wnt/β-catenin pathway in DLBCL cells. (A) Analysis of total β-catenin protein by Western blot analysis in LY8 cells transfected with negative control siRNA and MTDH siRNA. (B) and (C) Expression of cytoplasmic(B) and nuclear (C) β-catenin protein was analyzed in LY8 cells. The expression of β-actin in the cytoplasm and H3 in the nucleus served as controls for the efficiency of subcellular fractionation. Data expressed as mean±SD. *p<0.05 versus control; **p<0.01 versus control; ▴p>0.05 versus control.

To more immediately study the relationship between MTDH and Wnt/β-catenin signaling in DLBCL, we examined the expression of β-catenin in LY8 cells infected with MTDH-RNAi lentivirus or negative control lentivirus. The protein level of total β-catenin was decreased in MTDH-siRNA-treated cells (Figure 7A, P<0.01). At the same time, MTDH knockdown destabilized β-catenin protein and then resulted in a decrease in both cytoplasmic (Figure 7B, P<0.01) and nuclear β-catenin levels (Figure 7C, P<0.05).

Between the LY8 cells untreated and treated with TNF-α and siRNA towards MTDH, there was almost no change of β-catenin expression when the expression of MTDH was detected to be unchanged (Figure 5B, P>0.05). However, the protein level of total β-catenin was increased in the cells treated with TNF-α and control siRNA compared with the cells treated with TNF-α and MTDH siRNA (Figure 5B, P<0.05). The result probably suggested that the influence on β-catenin occurred after the expression change of MTDH rather than directly after TNF-α treatment.

These findings indicate that MTDH upregulation could directly or indirectly increase the protein level of β-catenin in the nucleus and thus play a significant part as an upstream activator for Wnt/β-catenin signaling pathway.

## Discussion

In recent years MTDH has been demonstrated as a potentially crucial mediator of various types of human malignancies. Its expression is significantly higher in melanoma, breast, esophageal, gastric, hepatocellular and prostate cancers, renal cell carcinoma, neuroblasoma and malignant glioma cell lines compared with their normal counterparts [12], [15]–[23]. These observations in cell lines have been confirmed in tumor patient specimens [15], [16], [18]–[27]. In the present manuscript we report that MTDH is highly expressed in DLBCL cell lines and patients with DLBCL for the first time. Our studies demonstrate that MTDH is overexpressed at both the mRNA and protein levels in DLBCL cell lines as well as DLBCL tissues. Besides, 76.67% (23/30) DLBCL samples showed upregulation of MTDH analyzed by immunohistochemical staining, while only little or rather non-expression of MTDH was detected in reactive hyperplasia of lymph node tissues. Further statistical analysis of the relationship between MTDH staining and the clinical features of DLBCL patients suggests that the over expression of MTDH is strongly correlated to the clinical staging of patients with DLBCL, whereas it is not correlated with the age, gender or B symptoms.

Studies are ongoing to further clarify the biological impact of MTDH overexpression on DLBCL tumor cells, in which we investigated gain-of-function through MTDH upregulation induced by TNF-α and loss-of-function through MTDH knockdown by small interfering RNA in DLBCL cells. We observed that the upregulation of MTDH in DLBCL cells could promote cell proliferation, which is in accordance with the inhibitory effect on cell apoptosis. Considering the possibility that the effect on the proliferation and apoptosis occurs directly after TNF-α treatment, we treated the cell lines with TNF-α and control siRNA or siRNA towards MTDH simultaneously and detected the apoptosis rate and β-catenin expression. The results suggested that there was almost no change of apoptosis rate and β-catenin expression between treated and untreated cells when the expression of MTDH was detected to be unchanged. However, cell apoptosis was inhibited and the protein level of total β-catenin was increased in the cells treated with TNF-α and control siRNA compared with the cells treated with TNF-α and MTDH siRNA. This probably suggested that the effect on the cell biological behavior and Wnt/β-catenin signaling occurred after the expression change of MTDH rather than directly after TNF-α treatment. The results also show that the reducing of MTDH in DLBCL cells could enhance cell apoptosis. Taken together, these data suggest that MTDH upregulation is likely associated with the pathogenesis of DLBCL.

Previous studies have demonstrated that MTDH promotes tumor initiation and progression by modulating multiple downstream oncogenic pathways, such as NF-κB, PI3K/Akt and Wnt/β-catenin pathways [28], [29]. It is known that activation of Wnt/β-catenin pathway is probably important for malignancy of DLBCL. β-catenin is the central transcription factor of the Wnt/β-catenin signaling pathway, and in certain cases it is accumulated in the nucleus and mediates the activation of Wnt/β-catenin pathway. In this present study, we validated the upregulation of total β-catenin and expression of nuclear β-catenin in DLBCL cell lines. Moreover, our research showed that MTDH upregulation elevates both total and nuclear β-catenin protein. The higher amount of β-catenin found in the nucleus could be a consequence of the increase in the total level of the protein or due to a specific role of MTDH in the translocation of β-catenin. That is to say MTDH upregulation promotes translocation of β-catenin to the nucleus directly or indirectly. In contrast, we showed the reduction of total, cytoplasmic and nuclear β-catenin protein induced by MTDH silencing, which revealed the close relationship between MTDH and Wnt/β-catenin pathway. These results indicate that MTDH plays a significant role as an upstream activator for Wnt/β-catenin signaling pathway in DLBCL. Recent studies illuminated that MTDH can activate Wnt/β-catenin signaling pathway via ERK42/44 activation, which then leads to GSK3β phosphorylation, and thus facilitate nuclear translocation of β-catenin in hepatocellular carcinoma [16]. In gastric cancer inhibition of MTDH may decrease the level of β-catenin and inactivate Wnt/β-catenin pathway through reducing phosphorylation of AKT and glycogen synthase kinase (GSK)-3β (Ser 9) [15]. To our knowledge, the mechanisms that MTDH actives Wnt/β-catenin pathway are not clear, accordingly further research should be done on the regulation of Wnt/β-catenin pathway by MTDH in human DLBCL. Scientists have revealed that some agents could lead to apoptosis or suppress the growth of lymphoma cells through Wnt/β-catenin signaling pathway [41], [44]. In addition, in two rituximab responsive DLBCL cell lines rituximab could affect the expression of genes in classical signaling cascades, including Wnt pathway, among others [42]. Since MTDH not only involves in the pathogenesis of DLBCL itself but also plays a significant role as an upstream activator for Wnt/β-catenin signaling pathway, there is great potential that can act as a therapeutic target of DLBCL.

In summary, our findings suggest that the over expression of MTDH is associated with the pathogenesis of DLBCL for the first time. We demonstrate that MTDH was markedly overexpressed in both DLBCL cell lines and tissues and β-catenin was upregulated, with its nuclear localization, in DLBCL cell lines compared with their counterparts. We found that the over expression of MTDH was strongly correlated to the clinical staging of patients with DLBCL. Furthermore, we determined that apoptosis of DLBCL cell lines was distinctly inhibited while cell proliferation was enhanced according with MTDH upregulation after treatment with TNF-α. Cell apoptosis was also promoted in MTDH knockdown cells in comparison to those transfected with negative control siRNA. Moreover, MTDH promotes growth and survival of DLBCL cells via regulating Wnt/β-catenin signaling pathway. The upregulation of MTDH induced by TNF-α could increase the protein level of total β-catenin and its nuclear translocation directly and indirectly, whereas, MTDH silencing could reduce the level of total β-catenin protein, facilitate the degradation of cytoplasmic β-catenin and decrease its nuclear translocation. Further efforts are needed to expound the particular molecular mechanism of MTDH upregulating Wnt/β-catenin pathway and understand the roles of MTDH in DLBCL development, which may enable MTDH to be a useful biomarker and potential therapeutic target for DLBCL.

## Materials and Methods

### Patient and Samples

Paraffin-embedded archived samples, including thirty cases of DLBCL diagnosed between January 2008 and December 2010 according to the WHO criteria [46], and fifteen reactive hyperplasia of lymph node tissues, were collected from the Shandong Provincial Hospital. Twenty-one biopsies of DLBCL tissues and twenty-five tissues from patients of reactive hyperplasia of lymph node were frozen and stored in liquid nitrogen until further use. Peripheral blood mononuclear cells (PBMCs) from healthy volunteers served as normal control compared with human DLBCL cell lines LY1 and LY8. Peripheral blood was collected by heparin anticoagulation and PBMCs were separated using the Ficoll-Hypaque gradient centrifugation method. The protocol was approved by the Shandong Provincial Hospital Ethics Committee and written informed consent was obtained from all participants involved in this study.

### Cell Lines and Cell Culture

All cell lines were maintained at 37°C in 5% carbon dioxide. The human DLBCL cell lines LY1 and LY8 were cultured in Iscove’s Modified Dulbecco’s Medium (IMDM; Hyclone, Logan, UT, USA) supplemented with 10% fetal bovine serum (FBS, HyClone, Logan, UT, USA) [47]. The 3 human MCL cell lines, Jeko-1,Mino,and SP53, were maintained in RPMI 1640 medium with L-glutamine (Hyclone, Logan, UT, USA), penicillin (100 U/mL), streptomycin (100 mg/mL) and 10%FBS as previously described [48].

### Antibodies and Reagents

Rabbit anti-MTDH polyclonal antibody was purchased from Invitrogen (Carlsbad, CA, USA). Rabbit anti-β-catenin polyclonal antibody and mouse anti-β-actin monoclonal antibody was from Abcam (Cambridge, MA). Rabbit anti-histone 3 (H3) polyclonal antibody was obtained from Beyotime (Shanghai, China). ^3^H-thymidine was purchased from Perkin-Elmer (Waltham, MA). TNF-α was purchased from PeproTech (Rocky Hill, NJ, USA). For studies, it was dissolved in 0.1% of BSA and aliquoted as a stock solution. To prepare working solutions, aliquots were further diluted in Iscove’s modified Dulbecco’s medium (IMDM; Hyclone, Logan, UT, USA) supplemented with 10% heat-inactivated fetal bovine serum (FBS, Hyclone, Logan, UT, USA) immediately before each experiment.

### Knockdown of Human MTDH by RNA Interference (RNAi)

RNAi target sequence to human MTDH gene was 5′-AACAGAAGAAGAAGAACCGGA-3′ by reference to the previous study [31]. The sequences of small interfering RNA (siRNA) targeting the human *MTDH* gene and negative control siRNA were cloned into the pGCL-GFP and generated using stable lentivirus expression vectors by Shanghai GeneChem. LY8 cells were plated in 96-well plates (10^4^cells/well) and were infected with MTDH-RNAi lentivirus or negative control lentivirus with the multiplicity of infection (MOI) 80 according to the manufacturer’s instructions. The medium was changed with fresh medium after 8–12 hours. Infection efficiencies were determined by GFP fluorescence. The cells were then harvested for mRNA and protein extraction or detection of cell apoptosis. In addition, the cells were incubated in the absence or presence of TNF-α for 48 hours (final concentration 250 pg/ml) and then harvested for protein extraction and detection of cell apoptosis.

**Table 2:** Primer Sequences.

Name	Sequence
MTDH-F	5′-TTACCACCGAGCAACTTACAAC-3′
MTDH-R	5′-ATTCCAGCCTCCTCCATTGAC -3′
β-catenin-F	5′-TGGCAGCAACAGTCTTACCT -3′
β-catenin-R	5′-CATAGCAGCTCGTACCCTCT -3′
β-actin-F	5′-TGACGTGGACATCCGCAAAG-3′
β-actin-R	5′-CTGGAAGGTGGACAGCGAGG-3′

### Reverse Transcription-Polymerase Chain Reaction (PCR) and Real-time Quantitative PCR

Total RNA was extracted from tissues using Trizol (Invitrogen). Then reverse transcription reaction was conducted by means of TaKaRa reverse transcription reagents (TaKaRa, Dalian, China). The reaction was incubated at 37°C for 15 minutes, and 85°C for 5 seconds. Amplification reactions were performed using SYBR Premix Ex Taq (Perfect Real Time) (TaKaRa, Dalian, China) on ABI 7500 Real-Time quantitative PCR System with cycling as follows: an initial cycle for 2 minutes at 95°C, followed by 40 bi-phasic cycles of 15 seconds at 95°C and 1 minute at 60°C. PCR products were confirmed as a single product at the desired size on agarose gels and visualized by ethidium bromide staining. Specific primers for RT-PCR were obtained from Biosune (Shanghai, China), and the primer sequences are listed in Table 2. Expression data were normalized to the geometric mean of housekeeping gene β-actin to control the variability in expression levels and analyzed using the 2^−△△CT^ method.

### Western Blot Analysis

Total protein was extracted from DLBCL tumors, reactive hyperplasia of lymph node tissues, LY1, LY8, Jeko-1, Mino, SP53 cell lines and PBMCs of healthy samples using RIPA and 1% PMSF (Shenergy Biocolor, Shanghai, China). For cytoplasmic and nuclear extracts, cells were washed with phosphate-buffered saline (PBS) and were lysed in NE-PER extraction reagent (Pierce) according to the manufacturer's protocol. The protein concentration of the samples was determined by the BCA assay (Shenergy Biocolor). Cell lysates were then electrophoresed on 10% SDS-polyacrylamide gels, while cytoplasmic and nuclear lysates were electrophoresed on 12% SDS-polyacrylamide gels, and next transferred onto nitrocellulose membranes. After the membranes were blocked with 5% skim milk in Tris-saline buffer with 0.1% Tween-20, they were subsequently probed with primary antibodies at 4°C overnight. After washings with TBST, secondary antibody conjugated with the horseradish peroxidase (Zhongshan Goldenbridge, Beijing, China) was added to the membranes. After washings with TBST, proteins were detected using the chemiluminescence detection kit (Millipore, Massachusmetts, USA). Antibodies used in this study included anti-MTDH (1 µg/ml), anti-β-catenin (1∶5000), anti-β-actin (1∶10000) and anti-H3 with a dilution of 1∶1000. Western blot results were analyzed using the Las-4000 Image software and Multi Gauge Ver.3.0 software (Fujifilm Life Science, Japan).

### Immunohistochemistry Study

In brief, formalin-fixed, paraffin-embedded tissue sections of 4-µM thickness were deparaffinized and hydrated. High-pressure antigen retrieval was performed using citrate buffer (pH6). Endogenous peroxidase was quenched with 3% hydrogen peroxide in methanol for 15 minutes, followed by incubation with normal serum to block non-specific staining. Rabbit anti-MTDH (1∶25) antibody was then incubated with the sections overnight at in a humidified chamber 4°C; the second antibody was from SP reagent kit (Zhongshan Goldenbridge Biotechnology Company, Beijing, China). After washing, the tissue sections were treated with biotinylated anti-rabbit secondary antibody, followed by further incubation with streptavidin-horseradish peroxidase complex. Stained with diaminobenzidine Kit (DAB, Zhongshan Goldenbridge Biotechnology Company, Beijing, China), the sections were counterstained with hematoxylin and mounted. Immunohistochemical staining of samples and negative controls occurred simultaneously, and the primary antibody was replaced with PBS for negative controls.

Immunohistochemical stainings were assessed in a series of randomly selected 5 high-power fields, which were believed to be representative of the average in tumors at×400 magnification, by two independent observers who were blinded to all clinical data. The sections were scored according to the proportion of positively stained tumor cells. Tumors displaying staining in 30% or more of the cells were categorized as positive cases. In the meanwhile, tumors displaying staining less than 30% of the cells were categorized as negative cases.

### Assessment of Cell Proliferation

LY1 and LY8 cell lines (5×10^3^, respectively) were seeded into 96-well plates, treated with TNF-α (250 pg/ml) and cultured for 48 hours. ^3^H-thymidine (1 µCi/well) was added into the cultures 16 hours before the end of the experiment. Then cell proliferation was evaluated using ^3^H-TdR incorporation method as described previously [49]. Triplicate wells were included in each sample.

### Assessment of Cell Apoptosis

After treatment of LY1 and LY8 cells with TNF-α (250 pg/ml) for 48 h, cell apoptosis and necrosis were determined using an annexin V- fluorescein isothiocyanate (FITC) and propidium iodide (PI) apoptosis detection kit (Neobioscience, Shenzhen, China), according to the manufacturer's instructions. Briefly, an aliquot of 10^6^ cells was incubated with annexin V-FITC and PI for 10 minutes at room temperature in the dark. Cells were then immediately analyzed with FACScan flow cytometer (Beckman Coulter, Chicago, USA). Viable cells are not stained with annexin V-FITC or PI. The necrotic cells were annexin V-FITC and PI-positive, whereas apoptotic cells were annexin V-FITC-positive and PI-negative [50], [51]. After infected with MTDH-RNAi lentivirus or negative control lentivirus, untreated or treated with TNF-α (250 pg/ml) for 48 hours, LY8 cell apoptosis was detected by using an annexin V- phycoerythrin (PE) and 7-amino-actinomycin D (7-AAD) apoptosis detection kit (MultiSciences Biotech Co., Ltd., Hangzhou, China), according to the manufacturer's instructions. Cells were also immediately detected with FACScan flow cytometer (Beckman Coulter, Chicago, USA). Viable cells are not stained with annexin V-PE or 7-AAD. The necrotic cells were annexin V-PE and 7-AAD-positive, whereas apoptotic cells were annexin V-PE-positive and 7-AAD-negative.

### Statistical Analysis

All statistical analyses were performed by using the statistics software SPSS 13.0 for Windows. The numerical data were statistically analyzed by 2-tailed Student’s t test t-test. Fisher’s exact test was used to analyze the relationship between the level of MTDH expression and clinicopathological features. Bivariate correlation between two independent variables was calculated by Spearman’s rank correlation coefficient. Statistically significance was defined as P<0.05.

## Supporting Information

Figure S1 **Infection efficiency of lentivirus and knockdown of MTDH in DLBCL cells.** (A) Infection efficiency of lentivirus in LY8 cells. The cells were treated with MTDH-specific lentivirus-mediated small interfering RNA (siMTDH) or with non-targeting siRNA as a negative control (siCon). Infection efficiency was observed by fluorescent microscopy: the upper row, bright field photos; the lower row, green fluorescence photos (original magnification ×200). (B) The knockdown efficiency of MTDH mRNA by siRNA was detected using quantitative PCR. The specific siRNA inhibited MTDH expression (p<0.05 versus control). (C) The specific siRNA effectively suppressed MTDH protein expression analyzed by Western blot. Expression of β-actin was used as loading control.
(TIF)
