# Supplementary figures and images for: Metadherin Contributes to the Pathogenesis of Diffuse Large B-cell Lymphoma
Source: PLoS One. 2012 Jun 29;7(6):e39449. doi: 10.1371/journal.pone.0039449 (PMC3387165; doi:10.1371/journal.pone.0039449)

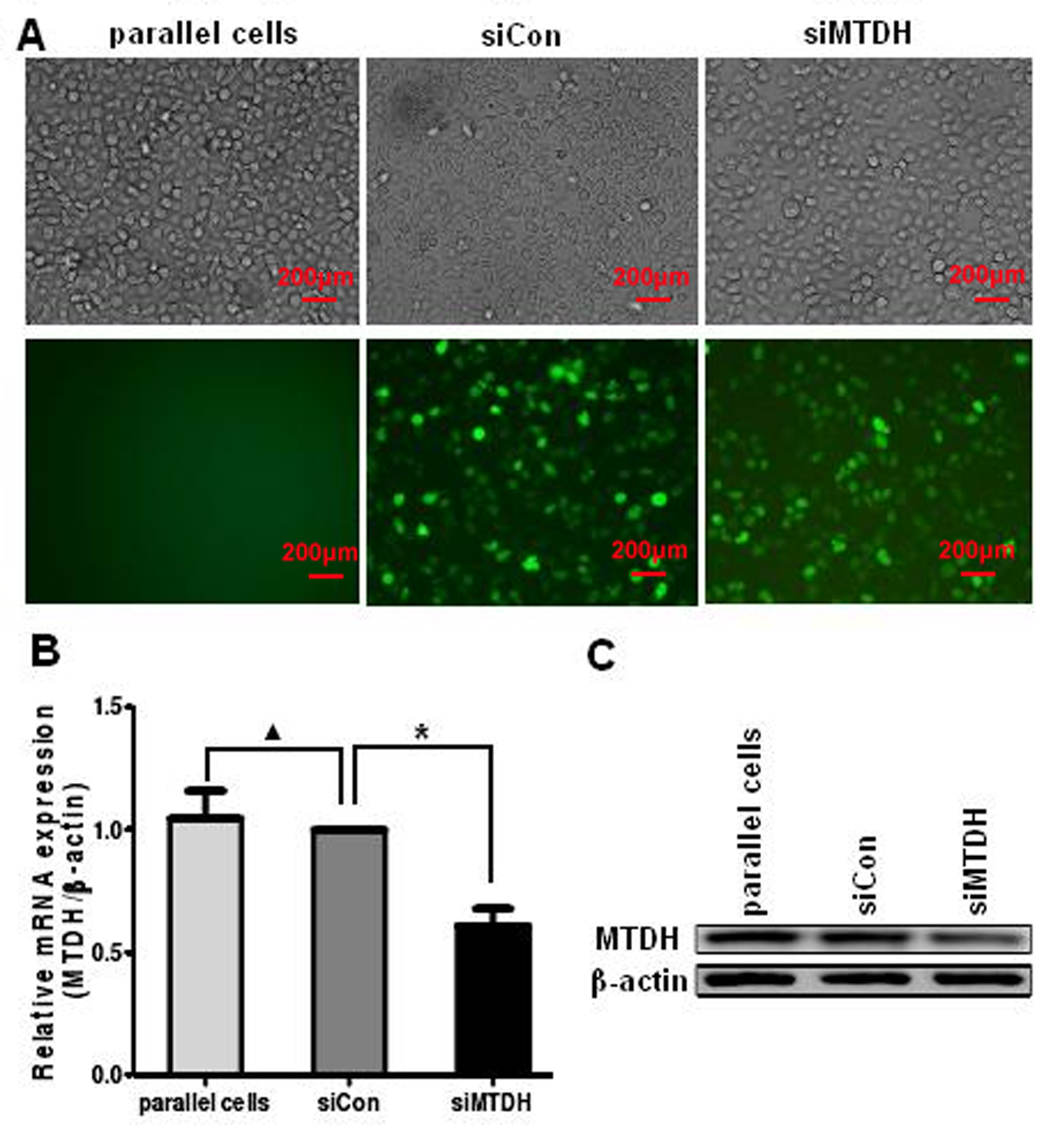

Supplement: Figure S1 — Infection efficiency of lentivirus and knockdown of MTDH in DLBCL cells. (A) Infection efficiency of lentivirus in LY8 cells. The cells were treated with MTDH-specific lentivirus-mediated small interfering RNA (siMTDH) or with non-targeting siRNA as a negative control (siCon). Infection efficiency was observed by fluorescent microscopy: the upper row, bright field photos; the lower row, green fluorescence photos (original magnification ×200). (B) The knockdown efficiency of MTDH mRNA by siRNA was detected using quantitative PCR. The specific siRNA inhibited MTDH expression (p<0.05 versus control). (C) The specific siRNA effectively suppressed MTDH protein expression analyzed by Western blot. Expression of β-actin was used as loading control. (TIF) [file pone.0039449.s001.tif]
